# Effect of Baking Conditions on Mycotoxin Levels in Flatbreads Prepared from Artificially Contaminated Doughs

**DOI:** 10.3390/foods14060910

**Published:** 2025-03-07

**Authors:** Kali Kotsiou, Michael A. Terzidis, Maria Papageorgiou

**Affiliations:** 1 Department of Food Science and Technology, International Hellenic University, Sindos Campus, 57400 Thessaloniki, Greece; kalikotsiou@food.ihu.gr; 2 Laboratory of Chemical Biology, Department of Nutritional Sciences and Dietetics, International Hellenic University, Sindos Campus, 57400 Thessaloniki, Greece; mterzidis@ihu.gr

**Keywords:** aflatoxins, ochratoxin A, zearalenone, deoxynivalenol, pita bread, baking effect, QuEChERS, LC-MS/MS analysis, multivariate analysis

## Abstract

This study investigated the impact of baking conditions on mycotoxin content in Greek pita bread, a single-layered flatbread baked at high temperatures for short time intervals. Dough samples were artificially contaminated with a multi-mycotoxin mixture, including aflatoxins (AFs) G2, G1, B2, and B1; ochratoxin A (OTA); deoxynivalenol (DON); and zearalenone (ZEA). Flatbreads were baked under three temperature–time combinations (220, 270, and 320 °C for 4, 2, and 1 min, respectively), with additional evaluation of a parbaking process (baking halted at 75% of the total time for each respective temperature, bread was stored at −18 °C, then bread was baked for 3 min at 180 °C). A QuEChERS-LC-MS/MS method was implemented for the determination of mycotoxins. The results demonstrated varying degrees of thermal degradation, with AFs B1 and G1 showing the highest decrease (39% on average), followed by AFG2, AFB2, ZEA, and DON (16–25%), while OTA remained relatively thermostable. Multivariate analyses classified flatbreads into two groups: higher baking temperatures and parbaking favored reductions in AFG1, AFG2, ZEA, OTA, and AFB2 levels while longer baking times at lower temperatures favored DON and AFB1 reduction. These findings provide insights for optimizing baking conditions to improve food safety in industrial and home-baking applications.

## 1. Introduction

Mycotoxins are secondary toxic metabolites produced by certain fungi that can contaminate crops and stored cereal grains and their presence in cereal-based products poses a significant food safety challenge globally [1]. Fusarium species are among the filamentous fungi with the most pronounced impact on agricultural production and human health as their mycotoxins not only infect crops, reducing yields, but also enter the food chain, posing health hazards to humans and animals [2]. To protect consumers from the toxic, mutagenic, and carcinogenic effects of mycotoxins, the European Union (EU) has established maximum levels for the individual mycotoxins in grains and their products. The different acceptable levels of mycotoxins established for flours and processed cereal-based foods reflect the change in mycotoxin levels that might occur during processing [3]. Among the over 300 mycotoxins that have been identified and reported, those that most regularly contaminate cereal grains and their products are aflatoxins (AF), ochratoxin A (OTA), zearalenone (ZEA), deoxynivalenol (DON), and T-2 toxin [4]. These compounds deserve special attention due to their high cytotoxicity, especially since mycotoxin combinations often exhibit stronger cytotoxic effects than individual mycotoxins [5]. Various food processing methods that can impact mycotoxin levels include sorting, trimming, cleaning, milling, brewing, cooking, baking, frying, roasting, canning, flaking, alkaline cooking, nixtamalization, and extrusion [4]. In the case of bakery products, some experimental findings suggest that mycotoxin levels might be reduced by controlling the kneading, proofing, fermentation, and baking steps [6]. The baking process can significantly impact mycotoxin stability; however, reported data remain controversial as they depend on the temperature–time combination, matrix effects, and sample size [6]. Most studies assess the impact of baking on only two to three mycotoxins, with DON and OTA being the most frequently studied. Numanoglu et al. [7] reported a temperature-dependent degradation of DON in a maize flour-based crust model. Similarly, baking cake analogs (3 g) for 40 min led to a DON decrease ranging from 29% at 140 °C to 81% at 200 °C [8]. However, to accurately evaluate the effects of baking temperature and duration on mycotoxin levels, the final product must maintain acceptable organoleptic properties. Thus, when measuring temperature effects in real samples, baking duration should be adjusted accordingly. Under this approach, during the rusk-making process in an industrial setting, baking duration appeared to be more effective in reducing DON levels compared to baking temperature when tested at 180–210 °C for 12–30 min [9]. This was attributed to the fact that, regardless of the oven temperature, the core temperature of the loaf never exceeded 100 °C. Instead, in the toasting step of rusk-making, temperature played a more significant role than duration. These findings, linked to heat penetration, also emphasize the importance of the surface-to-volume ratio. For example, substantial DON losses were recorded during the frying of turnover pie covers, reaching up to 66% and 28% when prepared with artificially and naturally contaminated flours, respectively [10]. Finally, a few studies have reported increases in mycotoxin levels, such as DON, in baked bread compared to the initially contaminated flour, which has been attributed to the release of DON from its conjugated forms during baking [11].

Generally, OTA demonstrates greater thermostability than DON, with some studies reporting no significant change in its content after baking normal-sized loaves [12,13,14]. However, reductions in OTA levels due to baking have been observed in certain cases that involve more intense thermal treatments. For instance, a 40% decrease was recorded in bread crusts after baking for 50 min at 190 °C [15]. Similarly, reductions between 21% and 64% were reported for cake analogs (3 g) baked for 40 min at temperatures ranging from 140 to 200 °C, respectively [8]. Compared to DON and OTA, fewer studies have investigated the stability of ZEA in bakery products and the reported effects of baking on its levels appear inconsistent as well. In maize bread, ZEA remained stable in the crumb but decreased by 13% in the crust after baking at 250 °C for 70 min [16]. In pan bread, baking at 180 °C for 25 min led to a significant ZEA decrease of 56–63% [17]. Differences in dough composition like sugar and flour content, along with temperature–time combinations, may also influence the outcomes. For instance, ZEA levels decreased by 27.5% in semisweet crackers baked at 245 °C for 5 min but remained unchanged in cracker biscuits baked at 285 °C for 3 min [12].

Regarding aflatoxins, limited research exists on the effect of baking conditions on bakery products. In the few available studies, the impact of baking is often assessed alongside other factors, such as dough fermentation conditions, rather than as an isolated parameter [18,19]. Noroozi, et al. [18] reported losses in AFB1 ranging from 62 to 89% during the baking of traditional Iranian breads. Similarly, Amra, et al. [20] observed reductions of approximately 47–57% for AFB1 and 44–55% for AFG1 after baking French breads, depending on the presence and type of preservatives used.

Discrepancies in the reported effects of baking on mycotoxins might arise from various methodological factors. Analytical methods can influence results while matrix changes due to processing may affect mycotoxin extractability and analysis. Additionally, dilution effects, experiment scale, and uneven mycotoxin distribution, especially in industrial settings, further complicate results interpretation [6]. Therefore, methodological optimization and careful data correction are essential to account for variability and measurement uncertainty. Most studies investigating the effect of baking conditions on mycotoxin levels in bakery products typically focus on one to three compounds. This is likely due to the complexity of extraction and detection methods or the high cost of standards when using spiking methods instead of naturally contaminated raw materials. However, mycotoxins from the same or different fungal species often co-occur in plant products, meaning cereals and their derivatives can be simultaneously contaminated with multiple mycotoxins [21]. Several analytical methods have been developed and validated for mycotoxin detection [22] but the heterogeneity of food matrices, along with the need for fast, simultaneous, and accurate determinations of multiple mycotoxins, present significant challenges for routine analysis [23]. Additionally, when analyzing whole processed foods, interactions between components and toxins or the possible formation of mycotoxin derivatives during processing can further complicate detection and quantification. To overcome the above-mentioned challenges, the most current research is directed to the development of analytical methods for simultaneous mycotoxin determination using fast, easy, and cheap extraction and clean-up procedures known as QuEChERS (quick, easy, cheap, effective, rugged, and safe) along with liquid chromatography-tandem mass spectrometry (LC-MA/MS) [24].

This study aimed to evaluate the impact of baking conditions on the extent of mycotoxin level reductions in Greek pita bread, a widely consumed single-layered flatbread traditionally baked at high temperatures for a short duration [25,26]. To achieve this, three different temperature–duration combinations were tested. Additionally, the effect of parbaking was evaluated as these products are either consumed after direct baking or undergo a prebaking and freezing process, requiring an additional baking step before consumption. The flatbreads were prepared from dough artificially contaminated with a multi-mycotoxin mixture consisting of AFs G2, G1, B2, and B1, OTA, DON, and ZEA. A QuEChERS-LC-MS/MS method was developed and evaluated for mycotoxin determination in the final products. By identifying the extent to which mycotoxins degrade or persist under specific baking conditions, this research aims to provide practical insights for improving food safety standards in both the baking industry and home-baking practices for this type of flatbread.

## 2. Materials and Methods

### 2.1. Materials

Methanol and water (LC-MS grade) were acquired from Fisher Scientific (Loughborough, UK). HPLC analytical standards: AFs (B1, B2, G1, G2) 10 μg/mL each in acetonitrile from RESTEK (Bellefonte, PA, USA), DON 1 mg from TRC (Toronto Research Chemicals Inc., Toronto, ON, Canada), ZEA 5 mg from Cayman Chemical (Ann Arbor, MI, USA), and OTA 10 μg/mL in acetonitrile from Merck KGaA (Darmstadt, Germany). Standards for contamination experiments: AF B1 (1 mg), AF B2 (1 mg), AF G1 (1 mg), and AF G2 (1 mg); DON (10 mg); OTA (1 mg); and ZEA (10 mg) were all obtained from TRC (Toronto Research Chemicals Inc., Toronto, ON, Canada). For flatbread preparation, wheat flour (WF) (70% of milling yield), instant baker’s yeast, and salt were purchased from a local market. FAL-QuEChERS^®^ CMS4 containing 0.62 g MgSO_4_, 0.15 g NaCl, 0.15 g trisodium citrate dihydrate (TCD), and 0.08 g disodium hydrogen citrate sesquihydrate (DHSQ) per 1 g were obtained from TÜV Austria Hellas (Athens, Greece).

### 2.2. Flatbread Preparation

Standard mycotoxins were used to contaminate the water used for dough preparation, with contamination levels (per kilogram of dough) for each mycotoxin set as follows: DON 500 μg/kg, AFs 10 μg/kg each, OTA 20 μg/kg, and ZEA 150 μg/kg. The flatbread doughs were prepared using WF, baker’s yeast (1%), salt (1%), and contaminated water (56%). After kneading the dough for 5 min in a farinograph mixer, it was divided into 30 g portions and proofed in a closed container at 30 °C for 30 min. Following proofing, the dough portions were shaped into round flatbreads with a diameter of 9.5 cm and immediately baked at 220, 270, or 320 °C for a period ranging from 1 to 4 min (direct baking). Parbaked flatbreads were also prepared by halting the baking process at 75% (prebaking) of the total time, storing them at −18 °C, and, after defrosting, baking for an additional 3 min at 180 °C (parbaking). The baking temperature–time combinations were determined through preliminary experiments to achieve a final product moisture content of approximately 35% (±2%), similar to that found in commercial products. Baking was conducted in an electric oven (Neff, Bruchsal, Germany), with the dough placed directly on the oven stone and flipped halfway through during both the direct and parbaking stages. Sampling was also conducted at the prebaking step to provide further insights into the potential degradation kinetics of mycotoxins, leading to a total of nine combinations of temperature–time sets (Table 1). The bread-making procedure was conducted in triplicate.

### 2.3. Extraction of Mycotoxins from Flatbreads

Mycotoxin extraction from flatbreads was performed using a QuEChERS-based method, an emerging technique for analyzing such substrates [24,27]. Initially, whole flatbreads were crumbled using a household food chopper/grinder to ensure homogeneity of the mycotoxin distribution. Then, 2 g of crumbled flatbread was placed in 15 mL falcon tubes, followed by the addition of 8 mL methanol (1% HCOOH). The sampling was performed in triplicate in each different baking batch. The mixture was vortexed for 30 s and repeated three times, with a 5-min rest between each vortexing. Following this step, the tubes were centrifuged at 6000 rpm for 5 min at 10 °C. A 3 mL aliquot of the supernatant was then cleaned using 2 g of extraction salts (FAL-QuEChERS^®^ CMS4), consisting of 1.25 g MgSO_4_, 0.30 g NaCl, 0.30 g TCD, and 0.15 g DHSQ. The salts were mixed with the sample for 20 min, with vortexing every 5 min for 30 s. A centrifugation step at 6000 rpm for 5 min was followed and 2 mL of the supernatant was collected and evaporated to dryness in a rotary evaporator (at 50 °C) and was placed at −18 °C until the analysis. The residue was reconstituted in 0.5 mL of a methanol–water mixture (50:50, *v*/*v*) and filtered via a 0.45 μm syringe filter prior to analysis.

### 2.4. Recovery Studies

A blank flatbread was prepared following the procedure described in Section 2.2, using clean water instead of contaminated water, to serve as a matrix for spiking in recovery studies. The spiking was performed within the following ranges: DON 100–1000 ng/g, AFs (B1, B2, G1, G2) 1–20 ng/g each, OTA 4–40 ng/g, and ZEN 30–300 ng/g. After spiking, the flatbreads were kept for 1 h with intermittent vortexing to ensure thorough diffusion of the mycotoxins within the matrix prior to extraction. Extraction was performed as described in Section 2.3 and mycotoxins were determined using LC-MS/MS with matrix-based calibration curves prepared from the same standards used in the contamination experiments as described in Section 2.6 below.

### 2.5. LC-MS/MS Analysis

The chromatographic analysis was carried out in an Agilent 1260 LC System (Agilent, Waltham, MA, USA). The analytes were resolved on a Zorbax XBD C18 (2.1 × 150 mm, 3.5 um) column at a flow rate of 0.3 mL/min. The injection volume was set to 5 μL. The mobile phase was composed of 0.1% formic acid (eluent A) and methanol containing 0.1% formic acid (eluent B). The elusion was performed using a gradient profile as follows: 95% A and 5% B at 0 min to 0.5 min, 30% A and 70% B at 7 min, 100% B at 9 min and kept constant up to 17 min, 95% A and 5% B at 17.1 min and kept constant up to 21 min for re-equilibration.

**Figure 1:**
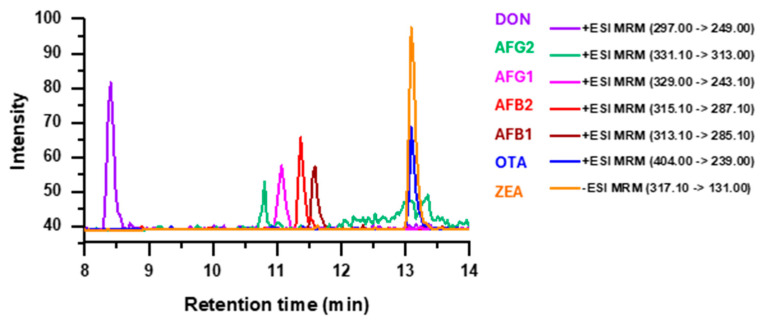
Representative LC−MS/MS selected ion monitoring (SIM) chromatogram of the analytes.

The MS analysis was performed on an Agilent 6400 Series Triple Quadrupole (Agilent, Waltham, MA, USA) using the following settings: gas temperature 350 °C; capillary 4000 V; gas flow 10 l/min; nebulizer 50 psi. The mass spectrometer was operated in multi-reaction monitoring (MRM) mode by monitoring three transitions (1 precursor ion, 3 product ions) for each compound with a dwell time of 100 ms (Table 2). One product ion was used for quantification (Figure 1) while the other two served for qualitative confirmation. The LC-MS/MS system, data acquisition, and processing were managed by MassHunter Workstation Software, Version B.06.00, supplied by Agilent Technologies.

**Figure 2:**
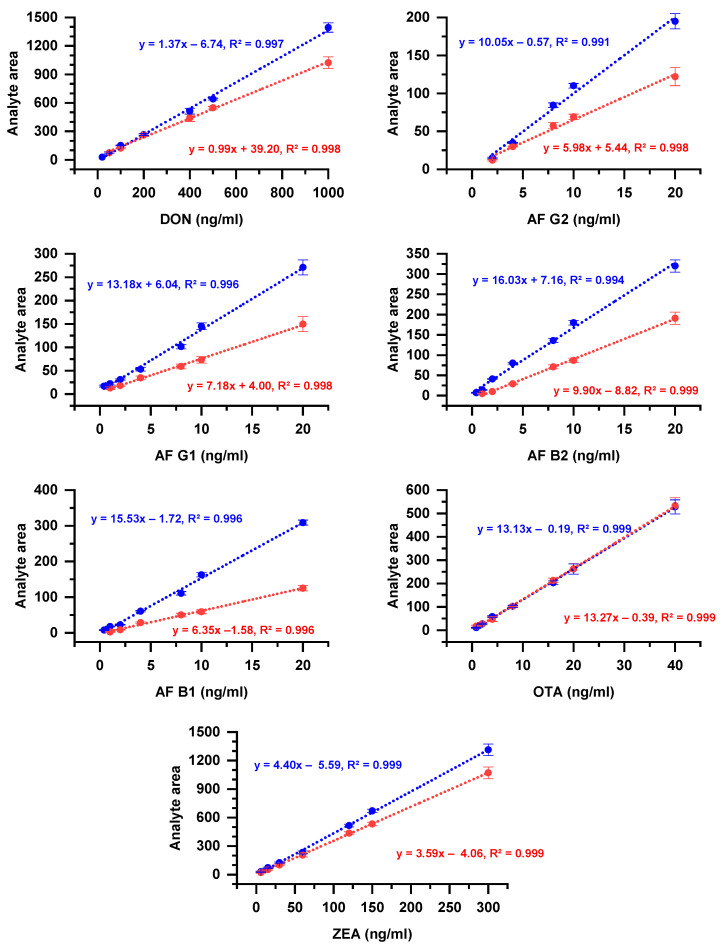
Comparison of solvent- and matrix-matched calibration curves. ●/∙∙∙∙∙, points and fitted linear regression curve based on solvent; ●/∙∙∙∙∙, points and fitted linear regression curve based on matrix.

The instrumental linearity, limit of detection (LOD), and limit of quantification (LOQ) were evaluated to ensure the method’s suitability for its specific application in the present study. Initially, the specificity was evaluated by analyzing blank extracts of baked flatbreads to verify the absence of interfering compounds at the retention time of the target analytes. The instrumental linearity was assessed by solvent calibration curves within the following ranges: DON 50–1000 ng/mL, AFs (B1, B2, G1, G2) 1–20 ng/mL each, OTA 2–40 ng/mL, and ZEA 6–300 ng/mL. Repeatability was assessed over two consecutive days by randomly injecting standard solutions throughout the sequence of extracted samples, as indicated by the standard deviations in Figure 2. The LOD and LOQ were calculated using the provided software as the lowest concentration of mycotoxin that produces a chromatographic peak at a signal-to-noise ratio (S/N) of 3 and 10, respectively. Under the applied method parameters, the LOQ values obtained for each mycotoxin were as follows: DON 50 mg/kg; AFs B1, B2, G1, and G2 all 1 μg/kg; OTA 2 mg/kg; and ZEA 6 mg/kg. The LOD values were calculated as follows: DON 20 mg/kg; AFs B1, B2, G1, and G2 all 0.4 μg/kg; OTA 0.8 mg/kg; and ZEA 3 mg/kg.

### 2.6. Mycotoxin Quantification in Flatbread and Data Expression

Matrix-based calibration curves were prepared using the same standards as in the contamination experiments, replacing the solvent with a blank flatbread extract. The following concentrations were prepared: DON 50–1000 ng/mL, AFs (B1, B2, G1, G2) 1–20 ng/mL each, OTA 2–40 ng/mL, and ZEA 6–300 ng/mL. Mycotoxin quantification was carried out using the matrix-based calibration curves, with recovery rates accounted for in the calculations. Each extract containing the mycotoxins was analyzed at least in triplicate to calculate the mean values of the individual mycotoxins. Standard solutions were injected regularly throughout the sequence of the extracted samples, allowing the standard curves to be frequently updated and ensuring the accuracy of the quantification. All analyses were performed over two consecutive days to fall within the tested period of repeatability, and the samples were analyzed in a random order. To ensure comparability, results were expressed on a dry basis. The percentage change in each mycotoxin was then determined by setting the initial contamination level (dry basis) as 100%.

### 2.7. Statistical Analysis

Data were analyzed using IBM SPSS Statistics, Version 29 (Armonk, NY, USA: IBM Corp.). Mean values of the analyzed parameters were compared using Tukey’s test at a significance level of α = 0.05. Principal component analysis (PCA) and hierarchical cluster analysis (HCA) were performed to identify potential relationships among mycotoxin levels in response to different baking conditions. The suitability of the dataset for PCA was assessed using the Kaiser–Meyer–Olkin (KMO) measure of sampling adequacy and Bartlett’s test of sphericity (Table S1). The KMO value was 0.56, indicating an acceptable level of sampling adequacy, while Bartlett’s test was significant (*p* = 0.014), confirming that the correlation matrix was not an identity matrix and that PCA was appropriate for data reduction and pattern recognition.

## 3. Results and Discussion

### 3.1. Extraction Procedure and LC-MS/MS Method Evaluation

The LC-MS/MS method linearity was evaluated using a least-squares regression line equation, yielding correlation coefficients ranging from 0.996 to 0.999, indicating a strong fit for all analytes. The method’s performance allowed for the detection of the studied mycotoxins at LOD and LOQ levels below the official limits established by the European Commission for cereal-derived products [3]. Since the applied method did not include a cleanup step, significant ion suppression effects were observed in the analyzed samples. This effect is illustrated in Figure 2 (regression analysis data are provided in Table S2), which compares solvent-based calibration curves to matrix-matched calibration curves at equivalent concentrations. The difference in slopes between these curves highlights the extent of matrix ion suppression for each investigated mycotoxin, with OTA being the least affected. In a relevant study, using a QuEChERS method, a similar effect was observed and it was attributed to the presence of endogenous substances in the sample that were retrieved in the final extract [24]. The addition of internal standards (isotopically labeled, deuterated, or analog) could minimize the variation among different samples by overcoming ion suppression/enhancement; however, due to the high cost of such standards, the use of external matrix-matched calibration has also been suggested in relevant studies [24,28]. Therefore, the matrix-matched calibration curves were used to quantify the analytes in our study.

**Figure 3:**
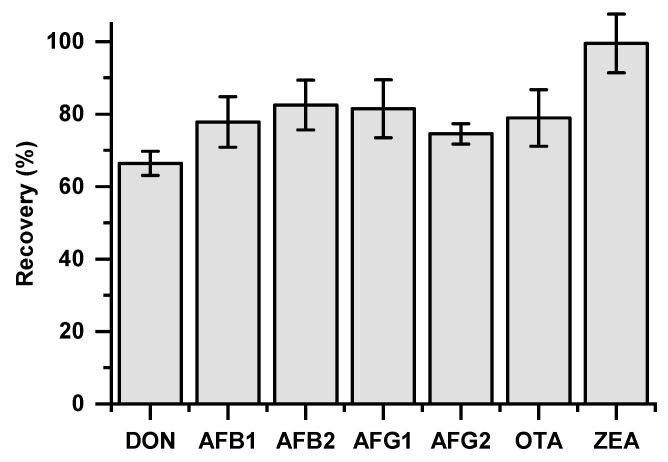
Average recoveries of mycotoxins within the studied ranges: DON 100–1000 μg/kg; AFs B1, B2, G1, G2 2–10 μg/kg each; OTA 5–40 μg/kg; and ZEN 30–300 μg/kg.

Following this procedure, recoveries of 66–100% (Figure 3) were achieved for the selected mycotoxins, aligning with ranges reported in the literature for similar extraction methods. For instance, recoveries between 64% and 100% have been documented for selected mycotoxins using 4 g MgSO_4_ and 1 g of NaCl in the extraction step [28]. For the concentrations examined in the recovery studies, the coefficients of variance were below 10%, indicating a consistent response for each mycotoxin within the selected contamination range—an essential factor for accurate data curation in our study. More specifically, the levels of mycotoxins used for the artificial contamination of dough were chosen based on the recovery studies and method evaluation considerations, ensuring that within the working ranges (i.e., initial contamination levels down to expected maximum reductions of 80%, as reported in the literature), the recovery percentages remained consistent and the acquired extracts fell within the range of the matrix-based standard curves used for quantification. Overall, the above methodology was developed and met the required criteria as part of the method’s intended application.

### 3.2. Effect of Baking Conditions on Mycotoxin Levels

**Figure 4:**
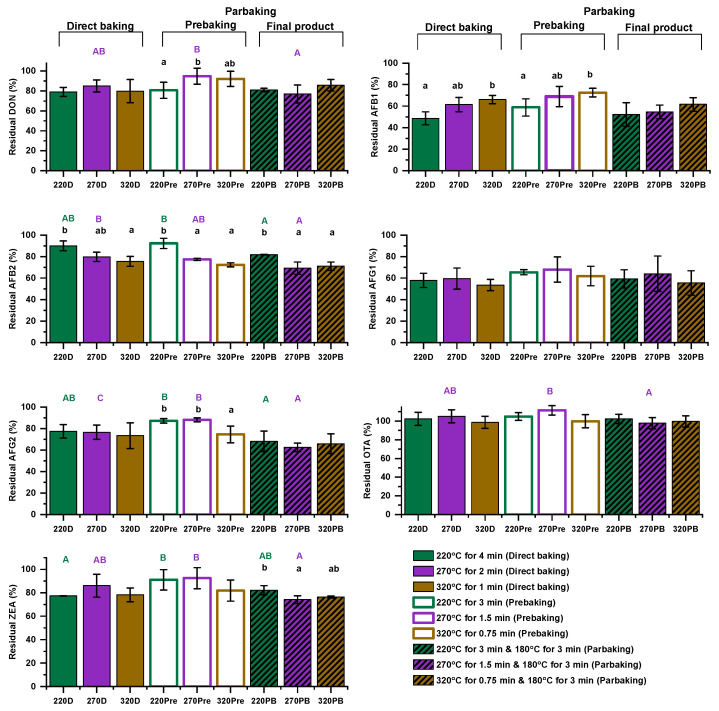
Residual percentages of individual mycotoxins in final and intermediate samples. Mean values for samples subjected to the same type of thermal treatment (direct, prebaked, or parbaked), e.g., 220D vs. 270D vs. 320D, are indicated with different lowercase letters when significantly different according to Tukey’s test (*p* < 0.05). Mean values for samples baked at the same temperature (220 °C, 270 °C, or 320 °C), e.g., 220D vs. 220Pre vs. 220PB, are indicated with different uppercase letters when significantly different according to Tukey’s test (*p* < 0.05). Only significant differences are displayed.

The impact of baking conditions on individual mycotoxin levels is illustrated in Figure 4 (statistical analysis is provided in Table S3). Mycotoxins were analyzed not only in the final baked products—both directly baked and parbaked—but also at the initial stage of the parbaking process (equivalent to 75% of the direct baking duration) to provide additional insights into possible degradation kinetics. In general, the results revealed that mycotoxins responded differently to the applied baking conditions. AFs G1 and B1 showed an average reduction of 39% while DON, ZEA, and AFs G2 and B2 exhibited average reductions ranging from 16% to 25%. In contrast, OTA appeared to be relatively thermostable. In the case of AFG1, all the applied baking conditions resulted in the same residual percentage; despite variations in the outcomes, no statistically significant differences among the applied conditions were observed. Regarding the directly baked flatbreads, the different combinations of time and temperature primarily affected AFs B1 and B2. Specifically, the AFB1 level showed a greater reduction when moving from samples baked at high temperature for short duration (320D) to those baked at low temperature for long duration (220D), whereas the opposite trend was observed for AFB2. This observation suggested that the duration of the process was the main factor influencing AFB1 level reduction, while temperature primarily affected AFB2. In parbaked flatbreads, AFB2 and ZEA showed similar patterns, with AFB2 levels being lower in the 270PB and 320PB samples, compared to 220PB, and ZEA being lower in 270PB than in 220PB. When comparing directly baked and parbaked flatbreads at the same temperature, the most notable differences were observed for the samples baked at the intermediate temperature of 270 °C, where AFs B2 and G2 were significantly lower in 270PB than in 270D. As previously mentioned, prebaked samples were included in the analysis, even though they were not considered final products, to gain further insights into potential degradation kinetics. When comparing baking duration at the same baking temperature (prebaked vs. directly baked samples), a shift toward lower residual levels in certain mycotoxins was observed, with AFG1 in 270Pre showing significant reduction compared to 270D. Notably, some prebaked samples exhibited a further reduction in mycotoxin levels during the second baking step (180 °C for 3 min), particularly 270PB, in which significant reductions in DON, AF G2, ZEA, and OTA were recorded compared to 270Pre. A similar effect was observed for AFs B2 and G2 in the 220PB and 220Pre samples. Overall, although temperature, duration, and parbaking influenced mycotoxin level reduction, identifying clear trends, especially in final products, remains challenging due to simultaneous adjustments in both temperature and duration for each treatment.

Several studies suggested that mycotoxins are influenced by high temperatures. The extent of their reduction during thermal food processing varies, depending on factors such as temperature, duration, product size, heat penetration, moisture content, certain matrix constituents, and pH while the type of mycotoxin plays a critical role in determining the level of reduction [29,30,31]. The above factors influencing the stability of mycotoxins, along with limitations in extraction and determination protocols, have led to somewhat controversial reported results. Furthermore, most studies do not account for dilution or concentration effects resulting from changes in moisture and composition, adding to the complexity of interpretation [6].

Regarding DON, a temperature dependence of its degradation rate was recorded in maize bread baking in the range of 150 to 250 °C [7]. Reduction of the DON level within the ranges of 4–14% [32] or 2–5% has been reported during wheat bread baking [33]. In comparison, the conditions applied in our study resulted in an average DON reduction of approximately 16%. The extent of the reduction in our case, despite the shorter baking duration, could be attributed to the higher baking temperatures applied to flatbreads compared to conventional bread baking, while the impact of the surface-to-volume ratio is also likely a significant contributing factor. Stadler, et al. [33] reported varying effects of baking conditions on DON reduction in biscuits, crackers, and breads, which were attributed to differences in pH, moisture content, and the surface-to-volume ratio of the individual baked products. The significance of surface-to-volume ratio and treatment intensity is further highlighted by the substantial decrease in DON content observed during the frying of turnover pie covers, reaching up to 66% and 28% when prepared with artificially and naturally contaminated flours, respectively [10].

Regarding AFs, most studies in the literature focus on evaluating the effect of fermentation on their reduction while the impact of baking conditions is typically assessed in conjunction with selected microorganisms as co-factors [18,19]. Therefore, no accurate comparisons can be made between the results reported in the literature and those obtained in this study. In an older study, AFB1 and AFG1 reductions of approximately 47–57% and 44–55%, respectively, were recorded in French bread baking, depending on the presence and type of preservatives used [20]. This study highlighted the similar behavior of AFB1 and AFG1 under baking conditions, a pattern that was also observed in our study, where both mycotoxins exhibited comparable reductions, with average losses of 39%. Moisture content plays a crucial role in reducing AFs during heating as it facilitates the hydrolysis of their lactone ring and, therefore, their degradation [34], which may explain the high reduction levels observed during the baking of bakery products.

Considering ZEA, Numanoglu, et al. [16] investigated its thermal degradation kinetics in a crust-like model made from maize flour and concluded that degradation occurred more rapidly as the baking temperature increased from 150 °C to 250 °C for the same baking duration. However, a similar conclusion could not be drawn in our study as increasing baking temperatures were accompanied by shorter baking times. Finally, OTA seemed to be quite stable under the baking conditions applied in our study, which is in accordance with previously reported data [13,14].

Overall, the data from our study suggest that baking temperature and time influence mycotoxin decrease. This effect was most pronounced for AFs B1 and G1, followed by AFG2, AFB2, ZEA, and DON, while OTA remained relatively thermostable. Among these, AFB2 showed the strongest dependency on baking conditions (temperature and/or time), followed by AFG2, ZEA, DON, and AFB1. The extent of individual mycotoxin decrease, even with a short baking duration, could be attributed to the high surface-to-volume ratio. Furthermore, the thin structure of the type of flatbread studied facilitated greater heat penetration during baking.

### 3.3. Principal Component Analysis and Hierarchical Cluster Analysis

**Figure 5:**
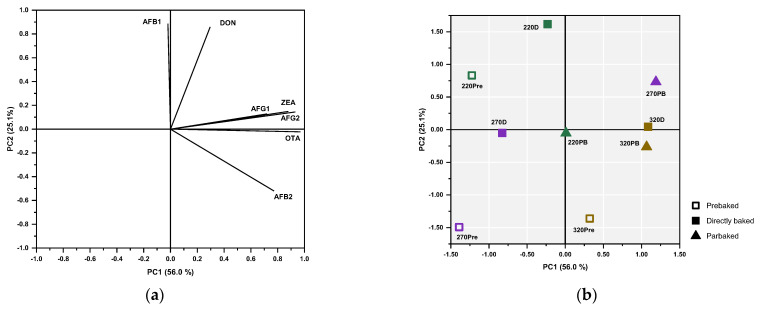
Component plot in rotated space (**a**) and sample distribution in the two-dimensional coordinate system (**b**) derived from principal component analysis (PCA); notation of samples as in Table 1.

PCA was conducted to identify potential relationships among mycotoxins decreases and the flatbreads baked under different conditions. Through this analysis, two components were extracted with an eigenvalue above 1, explaining 81.1% of the total variance (Table S1). AFs G1, G2, and B2; OTA; and ZEA were positively associated with PC1; on the other hand, AFB1 and DON were loaded onto PC2 (Figure 5a, Table S1). Therefore, flatbreads baked at 320 °C, either directly or via the parbaking process (320D and 320PB), along with 270PB, were strongly associated with higher reductions of AFG1, AFG2, ZEA, OTA, and AFB2, as reflected in their distribution along the *x*-axis (Figure 5b). Additionally, 320Pre was also located at the positive side of the *x*-axis.

The above analysis suggests that the decrease in AFG1, AFG2, ZEA, OTA, and AFB2 mycotoxins is primarily linked to the parbaking process or high-temperature exposure. On the other hand, 220D was the sample most correlated with DON and AFB1 reduction, showing a high projection on the positive side of the *y*-axis. In addition, 220Pre and 270PB were also positioned on the positive side of the *y*-axis, indicating that for DON and AFB1 reduction the duration of the process was more critical than the temperature level, as these samples underwent the longest treatment. Finally, some conclusions can be drawn from the distribution of the prebaked samples, which were scattered across the matrix. The decrease in mycotoxin content began during the prebaking step, following the previously described patterns of temperature versus duration response. Specifically, 220Pre (with the longest baking duration compared to 270Pre and 320Pre) was positioned on the positive side of the *y*-axis, correlating with a decrease in DON and AFB1. In contrast, 320Pre (subjected to the highest baking temperature among the prebaked samples) was located on the positive side of the *x*-axis, aligning with reductions in AFG1, AFG2, ZEA, OTA, and AFB2. Meanwhile, the intermediate treatment at 270Pre, with moderate temperature and duration, did not induce significant mycotoxin reductions.

**Figure 6:**
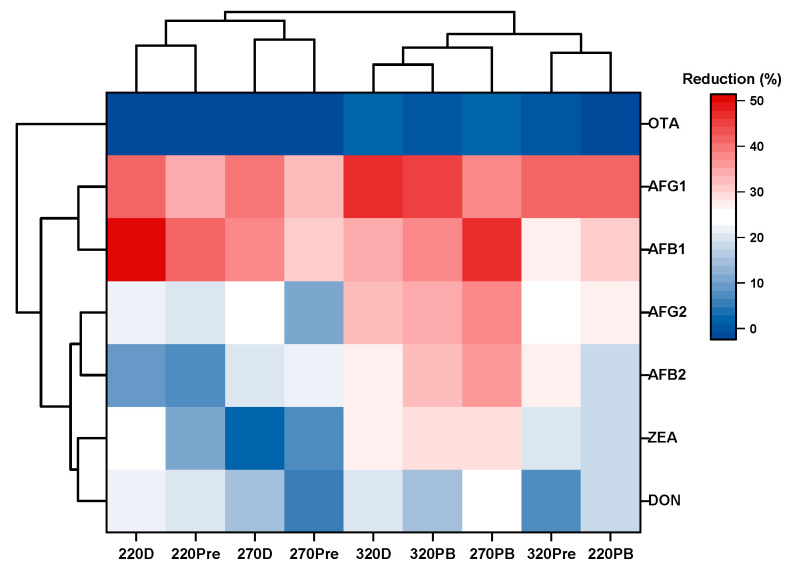
Hierarchical clustering heatmap of the extent of reduction (%) of individual mycotoxins in the flatbreads; notation of samples as in Table 1.

The observed effects are better illustrated in the heatmap and confirmed by HCA (Figure 6). As shown, apart from OTA, which, as expected, was clustered separately due to its stability to the applied conditions, the rest of the mycotoxins were grouped into two clusters: one consisting of AFs B1 and G1 and another comprising DON, ZEA, and AFs B2 and G2, indicating distinct responses to the applied baking conditions. Regarding flatbreads, they were categorized into two distinct clusters. Specifically, 220D, 220Pre, 270D, and 270Pre were grouped into the first cluster while 320D, 320PB, 270PB, 320Pre, and 220PB comprised the second cluster. This clustering further supported the results obtained from the PCA.

## 4. Conclusions

This study evaluated the impact of baking conditions on mycotoxin level reduction in Greek pita bread, focusing on different temperature–time combinations and the effect of parbaking. A QuEChERS-LC-MS/MS method was employed to accurately quantify mycotoxins in the final and intermediate products. The results showed that mycotoxins responded differently to the applied baking conditions, with AFs B1 and G1 exhibiting the highest reduction (average 39%), followed by AFG2, AFB2, ZEA, and DON (16–25%), while OTA remained relatively thermostable. Multivariate analyses, including PCA and HCA, helped clarify the relationships between baking conditions and mycotoxin reduction patterns. Excluding OTA, the mycotoxins clustered into two distinct groups: AFs B1 and G1, which were more susceptible to thermal treatment, and DON, ZEA, AFB2, and AFG2, which exhibited lower but still significant reductions under the examined baking conditions. Flatbreads were also classified into two clusters, reflecting the influence of temperature and duration. Considering the fully baked flatbreads (directly baked and prebaked), high-temperature baking and parbaking (320D, 320PB, 270PB) factors were associated with greater reductions in AFG1, AFG2, ZEA, OTA, and AFB2 whereas longer-duration treatments (220D, 270D) were more effective in reducing DON and AFB1. Although temperature, duration, and parbaking had an obvious effect on mycotoxin level reduction, clear trends were challenging to establish due to the interplay of these parameters. The use of artificially, highly contaminated doughs instead of naturally contaminated flour does not imply that WF exceeding EU regulatory limits can be considered safe for consumption after baking. However, given that such cereal-based products are widely consumed in many countries, optimizing processing conditions to minimize mycotoxin levels is crucial in reducing overall dietary exposure and preventing potential accumulation from multiple food sources. Therefore, these findings provide valuable insights for improving food safety in both industrial and home-baking applications.

## Figures and Tables

**Table 1:** Examined baking conditions and moisture contents of the final flatbread samples.

Sample Code	1st Baking	2nd Baking	Final Moisture (%)
220D	4 min/220 °C	-	34.6 (±1.8)
200Pre	3 min/220 °C	-	36.4 (±1.7)
220PB	3 min/220 °C	3 min/180 °C	33.3 (±1.2)
270D	2 min/270 °C	-	37.0 (±1.6)
270Pre	1.5 min/270 °C	-	37.9 (±1.7)
270PB	1.5 min/270 °C	3 min/180 °C	33.5 (±1.3)
320D	1 min/320 °C	-	37.3 (±1.7)
320Pre	45 s/320 °C	-	38.1 (±1.6)
320PB	45 s/320 °C	3 min/180 °C	35.2 (±1.5)

**Table 2:** LC-MS/MS acquisition parameters for mycotoxins.

Analyte	Retention Time (min)	Precursor Ion	Product Ion	Collision Energy (V)	Polarity
Deoxynivalenol (DON)	8.43	297	249 *	10	+
			231 **	14	+
			175 **	19	+
Aflatoxin B1 (AFB1)	11.61	313.1	285.1 *	23	+
			270 **	27	+
			241 **	37	+
Aflatoxin B2 (AFB2)	11.42	315.1	287.1 *	26	+
			271 **	32	+
			243 **	39	+
AflatoxinG1 (AFG1)	11.11	329	243.1 *	27	+
			215 **	32	+
			199 **	41	+
AflatoxinG2 (AFG2)	10.85	331.1	313 *	24	+
			285 **	28	+
			189 **	42	+
Ochratoxin A (OTA)	13.12	404	239 *	15	+
			358 **	24	+
			220.9 **	15	+
Zearalenone (ZEA)	13.16	317.1	131 *	19	-
			175 **	24	-
			273 **	29	-

* Quantitative ion. ** Qualitative ion.

## Data Availability

The original contributions presented in the study are included in the article/Supplementary Material, further inquiries can be directed to the corresponding author.

## Supplementary Materials

The following supporting information can be downloaded at: https://www.mdpi.com/article/10.3390/foods14060910/s1, Table S1: Principal component analysis output; Table S2: Regression analysis results corresponding to data presented in Figure 2; Table S3: Post hoc Tukey test results corresponding to data presented in Figure 3.

## Abbreviations

The following abbreviations are used in this manuscript:

AF	Aflatoxin
DON	Deoxynivalenol
OTA	Ochratoxin A
DOA	Deoxynivalenol
LOD	Limit of detection
LOQ	Limit ofquantification
QuEChERS	Quick, Easy, Cheap, Effective, Rugged, and Safe
