# Supplementary material for: Effect of Baking Conditions on Mycotoxin Levels in Flatbreads Prepared from Artificially Contaminated Doughs
Source: Foods. 2025 Mar 7;14(6):910. doi: 10.3390/foods14060910 (PMC11941633; doi:10.3390/foods14060910)
Supplement: Supplementary file 1 [file foods-14-00910-s001.zip › foods-3502288-supplementary.pdf]

Effect of baking conditions on mycotoxins levels in flatbreads prepared from artificially contaminated doughs

Kali Kotsiou [1], Michael A. Terzidis [2] and Maria Papageorgiou [1,*]

[1] Department of Food Science and Technology, International Hellenic University, Sindos campus, Thessaloniki,57400, Greece; kalikotsiou@food.ihu.gr (K.K), mariapapage@ihu.gr (M.P.)
[2] Laboratory of Chemical Biology, Department of Nutritional Sciences and Dietetics, International Hellenic University, Sindos campus, Thessaloniki, 57400, Greece; mterzidis@ihu.gr (M.A.T)
* Correspondence: mariapapage@ihu.gr; Tel.: +30-2310-013775

Supplementary material

Table S1. Principal component analysis output.

Correlation Matrix

		DON	AFB1	AFB2	AFG1	AFG2	OTA	ZEA
Correlation	DON	1.000	.678	-.158	.377	.551	.353	.409
	AFB1	.678	1.000	-.232	.026	.187	.071	.229
	AFB2	-.158	-.232	1.000	.258	.653	.684	.492
	AFG1	.377	.026	.258	1.000	.686	.625	.614
	AFG2	.551	.187	.653	.686	1.000	.876	.782
	OTA	.353	.071	.684	.625	.876	1.000	.916
	ZEA	.409	.229	.492	.614	.782	.916	1.000

KMO and Bartlett's Test

Kaiser-Meyer-Olkin Measure of Sampling Adequacy.		.564
Bartlett's Test of Sphericity	Approx. Chi-Square	37.687
	df	21
	Sig.	.014

Total Variance Explained

	Initial Eigenvalues			Extraction Sums of Squared Loadings			Rotation Sums of Squared Loadings[a]
Component	Total	% of Variance	Cumulative %	Total	% of Variance	Cumulative %	Total
1	3.916	55.945	55.945	3.916	55.945	55.945	3.846
2	1.758	25.117	81.062	1.758	25.117	81.062	1.943
3	.674	9.629	90.691				
4	.339	4.844	95.535				
5	.246	3.514	99.049				
6	.040	.574	99.623				
7	.026	.377	100.000				

Extraction Method: Principal Component Analysis.
a. When components are correlated, sums of squared loadings cannot be added to obtain a total variance.

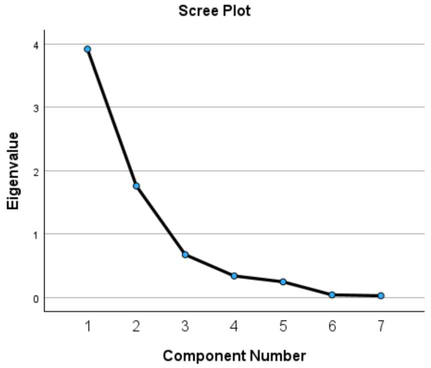

Pattern Matrix[a]

	Component 1	2
DON	.296	.859
AFB1	-.019	.886
AFB2	.772	-.520
AFG1	.720	.128
AFG2	.931	.144
OTA	.970	-.023
ZEA	.879	.149

Extraction Method: Principal Component Analysis.
Rotation Method: Oblimin with Kaiser Normalization.
a. Rotation converged in 5 iterations.

Table S2. Regression analysis results corresponding to data presented in Figure 2.

Equation	y = a + b*x	
Plot	Solvent-based	Matrix-based
**DON**		
Intercept	-6.73787 ± 14.8823	39.20202 ± 11.07557
Slope	1.37122 ± 0.03255	0.99413 ± 0.02243
Residual Sum of Squares	3851.15877	1245.55376
Pearson's r	0.99859	0.99898
R-Square (COD)	0.99719	0.99797
Adj. R-Square	0.99663	0.99746
**AFG2**		
Intercept	-0.56504 ± 6.20549	5.44309 ± 3.74115
Slope	10.05285 ± 0.57419	5.98374 ± 0.34617
Residual Sum of Squares	194.65041	70.74797
Pearson's r	0.99514	0.99502
R-Square (COD)	0.99031	0.99006
Adj. R-Square	0.98708	0.98675
**AFG1**		
Intercept	6.037 ± 3.62055	4 ± 1.53777
Slope	13.17711 ± 0.39599	7.17778 ± 0.15574
Residual Sum of Squares	227.92922	24.01111
Pearson's r	0.99775	0.99906
R-Square (COD)	0.9955	0.99812
Adj. R-Square	0.99461	0.99765
**AFB2**		
Intercept	7.15776 ± 4.94202	-8.81818 ± 1.8707
Slope	16.03294 ± 0.54053	9.90168 ± 0.18945
Residual Sum of Squares	424.67878	35.53356
Pearson's r	0.99717	0.99927
R-Square (COD)	0.99435	0.99854
Adj. R-Square	0.99322	0.99817
**AFB1**		
Intercept	-1.72082 ± 4.09709	-1.57576 ± 2.05295
Slope	15.52964 ± 0.44811	6.35084 ± 0.20791
Residual Sum of Squares	291.87886	42.7945
Pearson's r	0.99792	0.99786
R-Square (COD)	0.99585	0.99573
Adj. R-Square	0.99502	0.99466

**OTA**		
Intercept	-0.19224 ± 2.37624	-0.39697 ± 2.42484
Slope	13.13156 ± 0.12995	13.274 ± 0.13261
Residual Sum of Squares	98.1821	102.23903
Pearson's r	0.99976	0.99975
R-Square (COD)	0.99951	0.9995
Adj. R-Square	0.99941	0.9994
**ZEA**		
Intercept	-5.59435 ± 8.34324	-4.05912 ± 3.14365
Slope	4.39818 ± 0.06084	3.58724 ± 0.02292
Residual Sum of Squares	1210.37758	171.83847
Pearson's r	0.99952	0.9999
R-Square (COD)	0.99904	0.9998
Adj. R-Square	0.99885	0.99976

Table S3. Post-Hoc Tukey test results corresponding to data presented in Figure 3

- **220D vs 220Pre vs 220PB**

Multiple Comparisons

Tukey HSD

Dependent Variable	(I) Case	(J) Case	Mean Difference (I-J)	Std. Error	Sig.	95% Confidence Interval	
						Lower Bound	Upper Bound
DON %	220D	220PB	-2.0333	3.10686	.797	-11.5660	7.4994
		220Pre	-2.6667	3.10686	.684	-12.1994	6.8660
	220PB	220D	2.0333	3.10686	.797	-7.4994	11.5660
		220Pre	-.6333	3.10686	.977	-10.1660	8.8994
	220Pre	220D	2.6667	3.10686	.684	-6.8660	12.1994
		220PB	.6333	3.10686	.977	-8.8994	10.1660
AFB1 %	220D	220PB	-3.5667	6.71179	.859	-24.1603	17.0270
		220Pre	-10.2000	6.71179	.347	-30.7936	10.3936
	220PB	220D	3.5667	6.71179	.859	-17.0270	24.1603
		220Pre	-6.6333	6.71179	.610	-27.2270	13.9603
	220Pre	220D	10.2000	6.71179	.347	-10.3936	30.7936
		220PB	6.6333	6.71179	.610	-13.9603	27.2270
AFB2 %	220D	220PB	8.2000	3.13581	.088	-1.4215	17.8215
		220Pre	-2.3000	3.13581	.754	-11.9215	7.3215
	220PB	220D	-8.2000	3.13581	.088	-17.8215	1.4215
		220Pre	-10.5000*	3.13581	.036	-20.1215	-.8785
	220Pre	220D	2.3000	3.13581	.754	-7.3215	11.9215
		220PB	10.5000*	3.13581	.036	.8785	20.1215
AFG1 %	220D	220PB	-1.4333	5.19373	.959	-17.3691	14.5025
		220Pre	-7.6667	5.19373	.365	-23.6025	8.2691
	220PB	220D	1.4333	5.19373	.959	-14.5025	17.3691
		220Pre	-6.2333	5.19373	.495	-22.1691	9.7025
	220Pre	220D	7.6667	5.19373	.365	-8.2691	23.6025
		220PB	6.2333	5.19373	.495	-9.7025	22.1691
AFG2 %	220D	220PB	9.3333	5.47547	.279	-7.4669	26.1336
		220Pre	-9.7000	5.47547	.257	-26.5002	7.1002
	220PB	220D	-9.3333	5.47547	.279	-26.1336	7.4669
		220Pre	-19.0333*	5.47547	.031	-35.8336	-2.2331
	220Pre	220D	9.7000	5.47547	.257	-7.1002	26.5002
		220PB	19.0333*	5.47547	.031	2.2331	35.8336
OTA %	220D	220PB	.0000	2.86033	1.000	-8.7763	8.7763
		220Pre	-2.5667	2.86033	.661	-11.3429	6.2096
	220PB	220D	.0000	2.86033	1.000	-8.7763	8.7763
		220Pre	-2.5667	2.86033	.661	-11.3429	6.2096
	220Pre	220D	2.5667	2.86033	.661	-6.2096	11.3429
		220PB	2.5667	2.86033	.661	-6.2096	11.3429
ZEA %	220D	220PB	-4.9000	4.49543	.554	-18.6932	8.8932
		220Pre	-13.8000*	4.49543	.050	-27.5932	-.0068
	220PB	220D	4.9000	4.49543	.554	-8.8932	18.6932
		220Pre	-8.9000	4.49543	.198	-22.6932	4.8932
	220Pre	220D	13.8000*	4.49543	.050	.0068	27.5932
		220PB	8.9000	4.49543	.198	-4.8932	22.6932

Based on observed means.

The error term is Mean Square(Error) = 30.313.

*. The mean difference is significant at the .05 level.

Tukey HSD (Uses Harmonic Mean Sample Size = 3, alpha = .05)

DON %

Case	N	Subset
		1
220D	3	79.0000
220PB	3	81.0333
220Pre	3	81.6667
Sig.		.684

Means for groups in homogeneous subsets are displayed.
Based on observed means.
The error term is Mean Square (Error) = 14.479.

AFB1 %

Case	N	Subset
		1
220D	3	48.6333
220PB	3	52.2000
220Pre	3	58.8333
Sig.		.347

Means for groups in homogeneous subsets are displayed.
Based on observed means.
The error term is Mean Square (Error) = 67.572.

AFB2 %

Case	N	Subset	
		1	2
220PB	3	81.8333	
220D	3	90.0333	90.0333
220Pre	3		92.3333
Sig.		.088	.754

Means for groups in homogeneous subsets are displayed.
Based on observed means.
The error term is Mean Square (Error) = 14.750.

AFG1 %

Case	N	Subset
		1
220D	3	57.8333
220PB	3	59.2667
220Pre	3	65.5000
Sig.		.365

Means for groups in homogeneous subsets are displayed.
Based on observed means.
The error term is Mean Square (Error) = 40.462.

AFG2 %

Case	N	Subset	
		1	2
220PB	3	68.0667	
220D	3	77.4000	77.4000

220Pre	3		87.1000
Sig.		.279	.257

Means for groups in homogeneous subsets are displayed.
Based on observed means.
The error term is Mean Square (Error) = 44.971.

OTA %

		Subset
Case	N	1
220D	3	102.3333
220PB	3	102.3333
220Pre	3	104.9000
Sig.		.661

Means for groups in homogeneous subsets are displayed.
Based on observed means.
The error term is Mean Square (Error) = 12.272.

ZEA %

		Subset	
Case	N	1	2
220D	3	77.3000	
220PB	3	82.2000	82.2000
220Pre	3		91.1000
Sig.		.554	.198

Means for groups in homogeneous subsets are displayed.
Based on observed means.
The error term is Mean Square (Error) = 30.313.

- **270D vs 270Pre vs 270PB**

Multiple Comparisons

Tukey HSD

Dependent Variable	(I) Case	(J) Case	Mean Difference (I-J)	Std. Error	Sig.	95% Confidence Interval	
						Lower Bound	Upper Bound
DON %	270D	270PB	8.1000	4.96864	.305	-7.1452	23.3452
		270Pre	-10.1333	4.96864	.184	-25.3785	5.1118
	270PB	270D	-8.1000	4.96864	.305	-23.3452	7.1452
		270Pre	-18.2333*	4.96864	.024	-33.4785	-2.9882
	270Pre	270D	10.1333	4.96864	.184	-5.1118	25.3785
		270PB	18.2333*	4.96864	.024	2.9882	33.4785
AFB1 %	270D	270PB	6.8667	5.01206	.412	-8.5117	22.2450
		270Pre	-7.5000	5.01206	.357	-22.8784	7.8784
	270PB	270D	-6.8667	5.01206	.412	-22.2450	8.5117
		270Pre	-14.3667	5.01206	.064	-29.7450	1.0117
	270Pre	270D	7.5000	5.01206	.357	-7.8784	22.8784
		270PB	14.3667	5.01206	.064	-1.0117	29.7450
AFB2 %	270D	270PB	10.5667*	3.37880	.047	.1996	20.9338
		270Pre	2.4000	3.37880	.767	-7.9671	12.7671
	270PB	270D	-10.5667*	3.37880	.047	-20.9338	-.1996
		270Pre	-8.1667	3.37880	.113	-18.5338	2.2004
	270Pre	270D	-2.4000	3.37880	.767	-12.7671	7.9671
		270PB	8.1667	3.37880	.113	-2.2004	18.5338
AFG1 %	270D	270PB	-4.5000	10.58552	.907	-36.9793	27.9793
		270Pre	-8.4000	10.58552	.720	-40.8793	24.0793
	270PB	270D	4.5000	10.58552	.907	-27.9793	36.9793
		270Pre	-3.9000	10.58552	.929	-36.3793	28.5793
	270Pre	270D	8.4000	10.58552	.720	-24.0793	40.8793
		270PB	3.9000	10.58552	.929	-28.5793	36.3793
AFG2 %	270D	270PB	14.0667*	3.71444	.021	2.6698	25.4636
		270Pre	-11.5667*	3.71444	.047	-22.9636	-.1698
	270PB	270D	-14.0667*	3.71444	.021	-25.4636	-2.6698
		270Pre	-25.6333*	3.71444	.001	-37.0302	-14.2364
	270Pre	270D	11.5667*	3.71444	.047	.1698	22.9636
		270PB	25.6333*	3.71444	.001	14.2364	37.0302
OTA %	270D	270PB	7.3333	2.64015	.072	-.7674	15.4340
		270Pre	-6.3333	2.64015	.116	-14.4340	1.7674
	270PB	270D	-7.3333	2.64015	.072	-15.4340	.7674
		270Pre	-13.6667*	2.64015	.005	-21.7674	-5.5660
	270Pre	270D	6.3333	2.64015	.116	-1.7674	14.4340
		270PB	13.6667*	2.64015	.005	5.5660	21.7674
ZEA %	270D	270PB	12.1333	5.72693	.166	-5.4385	29.7051
		270Pre	-6.2000	5.72693	.558	-23.7718	11.3718
	270PB	270D	-12.1333	5.72693	.166	-29.7051	5.4385
		270Pre	-18.3333*	5.72693	.042	-35.9051	-.7615
	270Pre	270D	6.2000	5.72693	.558	-11.3718	23.7718
		270PB	18.3333*	5.72693	.042	.7615	35.9051

Based on observed means.

The error term is Mean Square(Error) = 49.197.

*. The mean difference is significant at the .05 level.

Tukey HSD (Uses Harmonic Mean Sample Size = 3, alpha = .05)

DON %

Case	N	Subset	
		1	2
270PB	3	77.0000	
270D	3	85.1000	85.1000
270Pre	3		95.2333
Sig.		.305	.184

Means for groups in homogeneous subsets are displayed.
Based on observed means.
The error term is Mean Square (Error) = 37.031.

AFB1 %

Case	N	Subset
		1
270PB	3	54.6333
270D	3	61.5000
270Pre	3	69.0000
Sig.		.064

Means for groups in homogeneous subsets are displayed.
Based on observed means.
The error term is Mean Square (Error) = 37.681.

AFB2 %

Case	N	Subset	
		1	2
270PB	3	69.2333	
270Pre	3	77.4000	77.4000
270D	3		79.8000
Sig.		.113	.767

Means for groups in homogeneous subsets are displayed.
Based on observed means.
The error term is Mean Square (Error) = 17.124.

AFG1 %

Case	N	Subset
		1
270D	3	59.6000
270PB	3	64.1000
270Pre	3	68.0000
Sig.		.720

Means for groups in homogeneous subsets are displayed.
Based on observed means.
The error term is Mean Square (Error) = 168.080.

AFG2 %

Case	N	Subset 1	2	3
270PB	3	62.5000		
270D	3		76.5667	
270Pre	3			88.1333
Sig.		1.000	1.000	1.000

Means for groups in homogeneous subsets are displayed.
Based on observed means.
The error term is Mean Square (Error) = 20.696.

OTA %

Case	N	Subset 1	2
270PB	3	97.8000	
270D	3	105.1333	105.1333
270Pre	3		111.4667
Sig.		.072	.116

Means for groups in homogeneous subsets are displayed.
Based on observed means.
The error term is Mean Square (Error) = 10.456.

ZEA %

Case	N	Subset 1	2
270PB	3	74.1333	
270D	3	86.2667	86.2667
270Pre	3		92.4667
Sig.		.166	.558

Means for groups in homogeneous subsets are displayed.
Based on observed means.
The error term is Mean Square (Error) = 49.197.

- **320D vs 320Pre vs 320PB**

Multiple Comparisons

Tukey HSD

Dependent Variable	(I) Case	(J) Case	Mean Difference (I-J)	Std. Error	Sig.	95% Confidence Interval	
						Lower Bound	Upper Bound
DON %	320D	320PB	-5.9667	6.83201	.675	-26.9291	14.9958
		320Pre	-12.4000	6.83201	.243	-33.3625	8.5625
	320PB	320D	5.9667	6.83201	.675	-14.9958	26.9291
		320Pre	-6.4333	6.83201	.636	-27.3958	14.5291
	320Pre	320D	12.4000	6.83201	.243	-8.5625	33.3625
		320PB	6.4333	6.83201	.636	-14.5291	27.3958
AFB1 %	320D	320PB	4.5333	3.94734	.522	-7.5782	16.6449
		320Pre	-6.4000	3.94734	.308	-18.5115	5.7115
	320PB	320D	-4.5333	3.94734	.522	-16.6449	7.5782
		320Pre	-10.9333	3.94734	.073	-23.0449	1.1782
	320Pre	320D	6.4000	3.94734	.308	-5.7115	18.5115
		320PB	10.9333	3.94734	.073	-1.1782	23.0449
AFB2 %	320D	320PB	4.5000	2.98068	.351	-4.6455	13.6455
		320Pre	3.2000	2.98068	.563	-5.9455	12.3455
	320PB	320D	-4.5000	2.98068	.351	-13.6455	4.6455
		320Pre	-1.3000	2.98068	.902	-10.4455	7.8455
	320Pre	320D	-3.2000	2.98068	.563	-12.3455	5.9455
		320PB	1.3000	2.98068	.902	-7.8455	10.4455
AFG1 %	320D	320PB	-1.9000	7.25156	.963	-24.1498	20.3498
		320Pre	-8.3667	7.25156	.520	-30.6165	13.8831
	320PB	320D	1.9000	7.25156	.963	-20.3498	24.1498
		320Pre	-6.4667	7.25156	.665	-28.7165	15.7831
	320Pre	320D	8.3667	7.25156	.520	-13.8831	30.6165
		320PB	6.4667	7.25156	.665	-15.7831	28.7165
AFG2 %	320D	320PB	7.5000	7.99152	.638	-17.0202	32.0202
		320Pre	-1.1000	7.99152	.990	-25.6202	23.4202
	320PB	320D	-7.5000	7.99152	.638	-32.0202	17.0202
		320Pre	-8.6000	7.99152	.561	-33.1202	15.9202
	320Pre	320D	1.1000	7.99152	.990	-23.4202	25.6202
		320PB	8.6000	7.99152	.561	-15.9202	33.1202
OTA %	320D	320PB	-.9667	3.28374	.954	-11.0421	9.1088
		320Pre	-1.1667	3.28374	.934	-11.2421	8.9088
	320PB	320D	.9667	3.28374	.954	-9.1088	11.0421
		320Pre	-.2000	3.28374	.998	-10.2754	9.8754
	320Pre	320D	1.1667	3.28374	.934	-8.9088	11.2421
		320PB	.2000	3.28374	.998	-9.8754	10.2754
ZEA %	320D	320PB	1.8667	5.08695	.929	-13.7415	17.4748
		320Pre	-3.6333	5.08695	.765	-19.2415	11.9748
	320PB	320D	-1.8667	5.08695	.929	-17.4748	13.7415
		320Pre	-5.5000	5.08695	.559	-21.1082	10.1082
	320Pre	320D	3.6333	5.08695	.765	-11.9748	19.2415
		320PB	5.5000	5.08695	.559	-10.1082	21.1082

Based on observed means.

The error term is Mean Square (Error) = 38.816.

Tukey HSD (Uses Harmonic Mean Sample Size = 3, alpha = .05)

DON %

Case	N	Subset
		1
320D	3	79.8000
320PB	3	85.7667
320Pre	3	92.2000
Sig.		.243

Means for groups in homogeneous subsets are displayed.
Based on observed means.
The error term is Mean Square (Error) = 70.014.

AFB1 %

Case	N	Subset
		1
320PB	3	61.6000
320D	3	66.1333
320Pre	3	72.5333
Sig.		.073

Means for groups in homogeneous subsets are displayed.
Based on observed means.
The error term is Mean Square (Error) = 23.372.

AFB2 %

Case	N	Subset
		1
320PB	3	71.1000
320Pre	3	72.4000
320D	3	75.6000
Sig.		.351

Means for groups in homogeneous subsets are displayed.
Based on observed means.
The error term is Mean Square (Error) = 13.327.

AFG1 %

Case	N	Subset
		1
320D	3	53.6000
320PB	3	55.5000
320Pre	3	61.9667
Sig.		.520

Means for groups in homogeneous subsets are displayed.
Based on observed means.
The error term is Mean Square (Error) = 78.878.

AFG2 %

Case	N	Subset
		1
320PB	3	65.9000
320D	3	73.4000
320Pre	3	74.5000
Sig.		.561

Means for groups in homogeneous subsets are displayed.
Based on observed means.
The error term is Mean Square (Error) = 95.797.

OTA %

Case	N	Subset<br>1
320D	3	98.6333
320PB	3	99.6000
320Pre	3	99.8000
Sig.		.934

Means for groups in homogeneous subsets are displayed.
Based on observed means.
The error term is Mean Square (Error) = 16.174.

ZEA %

Case	N	Subset<br>1
320PB	3	76.3333
320D	3	78.2000
320Pre	3	81.8333
Sig.		.559

Means for groups in homogeneous subsets are displayed.
Based on observed means.
The error term is Mean Square (Error) = 38.816.

➢ 220Pre vs 270Pre vs 320Pre

Multiple Comparisons

Tukey HSD

Dependent Variable	(I) Case	(J) Case	Mean Difference (I-J)	Std. Error	Sig.	95% Confidence Interval Lower Bound	Upper Bound
DON %	220Pre	270Pre	-13.5667*	4.32092	.046	-26.8244	-.3089
		320Pre	-10.5333	4.32092	.111	-23.7911	2.7244
	270Pre	220Pre	13.5667*	4.32092	.046	.3089	26.8244
		320Pre	3.0333	4.32092	.771	-10.2244	16.2911
	320Pre	220Pre	10.5333	4.32092	.111	-2.7244	23.7911
		270Pre	-3.0333	4.32092	.771	-16.2911	10.2244
AFB1 %	220Pre	270Pre	-10.1667	4.41378	.131	-23.7094	3.3760
		320Pre	-13.7000*	4.41378	.048	-27.2427	-.1573
	270Pre	220Pre	10.1667	4.41378	.131	-3.3760	23.7094
		320Pre	-3.5333	4.41378	.716	-17.0760	10.0094
	320Pre	220Pre	13.7000*	4.41378	.048	.1573	27.2427
		270Pre	3.5333	4.41378	.716	-10.0094	17.0760
AFB2 %	220Pre	270Pre	14.9333*	2.41584	.002	7.5209	22.3458
		320Pre	19.9333*	2.41584	<.001	12.5209	27.3458
	270Pre	220Pre	-14.9333*	2.41584	.002	-22.3458	-7.5209
		320Pre	5.0000	2.41584	.177	-2.4125	12.4125
	320Pre	220Pre	-19.9333*	2.41584	<.001	-27.3458	-12.5209
		270Pre	-5.0000	2.41584	.177	-12.4125	2.4125
AFG1 %	220Pre	270Pre	-2.5000	7.10091	.935	-24.2876	19.2876
		320Pre	3.5333	7.10091	.875	-18.2542	25.3209
	270Pre	220Pre	2.5000	7.10091	.935	-19.2876	24.2876
		320Pre	6.0333	7.10091	.689	-15.7542	27.8209
	320Pre	220Pre	-3.5333	7.10091	.875	-25.3209	18.2542
		270Pre	-6.0333	7.10091	.689	-27.8209	15.7542
AFG2 %	220Pre	270Pre	-1.0333	3.86216	.962	-12.8835	10.8168
		320Pre	12.6000*	3.86216	.039	.7498	24.4502
	270Pre	220Pre	1.0333	3.86216	.962	-10.8168	12.8835
		320Pre	13.6333*	3.86216	.029	1.7832	25.4835
	320Pre	220Pre	-12.6000*	3.86216	.039	-24.4502	-.7498
		270Pre	-13.6333*	3.86216	.029	-25.4835	-1.7832
OTA %	220Pre	270Pre	-6.5667	2.63453	.103	-14.6501	1.5168
		320Pre	5.1000	2.63453	.209	-2.9835	13.1835
	270Pre	220Pre	6.5667	2.63453	.103	-1.5168	14.6501
		320Pre	11.6667*	2.63453	.011	3.5832	19.7501
	320Pre	220Pre	-5.1000	2.63453	.209	-13.1835	2.9835
		270Pre	-11.6667*	2.63453	.011	-19.7501	-3.5832
ZEA %	220Pre	270Pre	-1.3667	7.16002	.980	-23.3356	20.6022
		320Pre	9.2667	7.16002	.448	-12.7022	31.2356
	270Pre	220Pre	1.3667	7.16002	.980	-20.6022	23.3356
		320Pre	10.6333	7.16002	.362	-11.3356	32.6022
	320Pre	220Pre	-9.2667	7.16002	.448	-31.2356	12.7022
		270Pre	-10.6333	7.16002	.362	-32.6022	11.3356

Based on observed means.

The error term is Mean Square (Error) = 76.899.

*. The mean difference is significant at the .05 level.

Tukey HSD (Uses Harmonic Mean Sample Size = 3, alpha = .05)

DON %

Case	N	Subset	
		1	2
220Pre	3	81.6667	
320Pre	3	92.2000	92.2000
270Pre	3		95.2333
Sig.		.111	.771

Means for groups in homogeneous subsets are displayed.
Based on observed means.
The error term is Mean Square(Error) = 28.006.

AFB1 %

Case	N	Subset	
		1	2
220Pre	3	58.8333	
270Pre	3	69.0000	69.0000
320Pre	3		72.5333
Sig.		.131	.716

Means for groups in homogeneous subsets are displayed.
Based on observed means.
The error term is Mean Square(Error) = 29.222.

AFB2 %

Case	N	Subset	
		1	2
320Pre	3	72.4000	
270Pre	3	77.4000	
220Pre	3		92.3333
Sig.		.177	1.000

Means for groups in homogeneous subsets are displayed.
Based on observed means.
The error term is Mean Square(Error) = 8.754.

AFG1 %

Case	N	Subset
		1
320Pre	3	61.9667
220Pre	3	65.5000
270Pre	3	68.0000
Sig.		.689

Means for groups in homogeneous subsets are displayed.
Based on observed means.
The error term is Mean Square(Error) = 75.634.

AFG2 %

Case	N	Subset	
		1	2
320Pre	3	74.5000	
220Pre	3		87.1000
270Pre	3		88.1333
Sig.		1.000	.962

Means for groups in homogeneous subsets are displayed.
Based on observed means.
The error term is Mean Square(Error) = 22.374.

OTA %

Case	N	Subset	
		1	2
320Pre	3	99.8000	
220Pre	3	104.9000	104.9000
270Pre	3		111.4667
Sig.		.209	.103

Means for groups in homogeneous subsets are displayed.
Based on observed means.
The error term is Mean Square(Error) = 10.411.

ZEA %

Case	N	Subset
		1
320Pre	3	81.8333
220Pre	3	91.1000
270Pre	3	92.4667
Sig.		.362

Means for groups in homogeneous subsets are displayed.
Based on observed means.
The error term is Mean Square(Error) = 76.899.

➢ 220D vs 270D vs 320D

Multiple Comparisons

Tukey HSD

Dependent Variable	(I) Case	(J) Case	Mean Difference (I-J)	Std. Error	Sig.	95% Confidence Interval	
						Lower Bound	Upper Bound
DON %	220D	270D	-6.1000	6.51170	.639	-26.0797	13.8797
		320D	-.8000	6.51170	.992	-20.7797	19.1797
	270D	220D	6.1000	6.51170	.639	-13.8797	26.0797
		320D	5.3000	6.51170	.709	-14.6797	25.2797
	320D	220D	.8000	6.51170	.992	-19.1797	20.7797
		270D	-5.3000	6.51170	.709	-25.2797	14.6797
AFB1 %	220D	270D	-12.8667	4.59436	.070	-26.9634	1.2301
		320D	-17.5000*	4.59436	.021	-31.5968	-3.4032
	270D	220D	12.8667	4.59436	.070	-1.2301	26.9634
		320D	-4.6333	4.59436	.599	-18.7301	9.4634
	320D	220D	17.5000*	4.59436	.021	3.4032	31.5968
		270D	4.6333	4.59436	.599	-9.4634	18.7301
AFB2 %	220D	270D	10.2333	3.71613	.074	-1.1688	21.6354
		320D	14.4333*	3.71613	.019	3.0312	25.8354
	270D	220D	-10.2333	3.71613	.074	-21.6354	1.1688
		320D	4.2000	3.71613	.532	-7.2021	15.6021
	320D	220D	-14.4333*	3.71613	.019	-25.8354	-3.0312
		270D	-4.2000	3.71613	.532	-15.6021	7.2021
AFG1 %	220D	270D	-1.7667	6.09742	.955	-20.4752	16.9419
		320D	4.2333	6.09742	.775	-14.4752	22.9419
	270D	220D	1.7667	6.09742	.955	-16.9419	20.4752
		320D	6.0000	6.09742	.612	-12.7086	24.7086
	320D	220D	-4.2333	6.09742	.775	-22.9419	14.4752
		270D	-6.0000	6.09742	.612	-24.7086	12.7086
AFG2 %	220D	270D	.8333	7.05287	.992	-20.8068	22.4735
		320D	4.0000	7.05287	.842	-17.6401	25.6401
	270D	220D	-.8333	7.05287	.992	-22.4735	20.8068
		320D	3.1667	7.05287	.897	-18.4735	24.8068
	320D	220D	-4.0000	7.05287	.842	-25.6401	17.6401
		270D	-3.1667	7.05287	.897	-24.8068	18.4735
OTA %	220D	270D	-2.8000	3.85775	.758	-14.6366	9.0366
		320D	3.7000	3.85775	.626	-8.1366	15.5366
	270D	220D	2.8000	3.85775	.758	-9.0366	14.6366
		320D	6.5000	3.85775	.285	-5.3366	18.3366
	320D	220D	-3.7000	3.85775	.626	-15.5366	8.1366
		270D	-6.5000	3.85775	.285	-18.3366	5.3366
ZEA %	220D	270D	-8.9667	4.64144	.210	-23.2079	5.2745
		320D	-.9000	4.64144	.980	-15.1412	13.3412
	270D	220D	8.9667	4.64144	.210	-5.2745	23.2079
		320D	8.0667	4.64144	.267	-6.1745	22.3079
	320D	220D	.9000	4.64144	.980	-13.3412	15.1412
		270D	-8.0667	4.64144	.267	-22.3079	6.1745

Based on observed means.

The error term is Mean Square(Error) = 32.314.

*. The mean difference is significant at the .05 level.

Tukey HSD (Uses Harmonic Mean Sample Size = 3, alpha = .05)

DON %

Case	N	Subset
		1
220D	3	79.0000
320D	3	79.8000
270D	3	85.1000
Sig.		.639

Means for groups in homogeneous subsets are displayed.
Based on observed means.
The error term is Mean Square (Error) = 63.603.

AFB1 %

Case	N	Subset	
		1	2
220D	3	48.6333	
270D	3	61.5000	61.5000
320D	3		66.1333
Sig.		.070	.599

Means for groups in homogeneous subsets are displayed.
Based on observed means.
The error term is Mean Square (Error) = 31.662.

AFB2 %

Case	N	Subset	
		1	2
320D	3	75.6000	
270D	3	79.8000	79.8000
220D	3		90.0333
Sig.		.532	.074

Means for groups in homogeneous subsets are displayed.
Based on observed means.
The error term is Mean Square (Error) = 20.714.

AFG1 %

Case	N	Subset
		1
320D	3	53.6000
220D	3	57.8333
270D	3	59.6000
Sig.		.612

Means for groups in homogeneous subsets are displayed.
Based on observed means.
The error term is Mean Square (Error) = 55.768.

AFG2 %

Case	N	Subset
		1
320D	3	73.4000
270D	3	76.5667
220D	3	77.4000
Sig.		.842

Means for groups in homogeneous subsets are displayed.
Based on observed means.
The error term is Mean Square (Error) = 74.614.

OTA %

Case	N	Subset
		1
320D	3	98.6333
220D	3	102.3333
270D	3	105.1333
Sig.		.285

Means for groups in homogeneous subsets are displayed.
Based on observed means.
The error term is Mean Square (Error) = 22.323.

ZEA %

Case	N	Subset
		1
220D	3	77.3000
320D	3	78.2000
270D	3	86.2667
Sig.		.210

Means for groups in homogeneous subsets are displayed.
Based on observed means.
The error term is Mean Square(Error) = 32.314.

➢ 220PB vs 270PB vs 320PB

Multiple Comparisons

Tukey HSD

Dependent Variable	(I) Case	(J) Case	Mean Difference (I-J)	Std. Error	Sig.	95% Confidence Interval Lower Bound	95% Confidence Interval Upper Bound
DON %	220PB	270PB	4.0333	4.46584	.658	-9.6691	17.7357
		320PB	-4.7333	4.46584	.570	-18.4357	8.9691
	270PB	220PB	-4.0333	4.46584	.658	-17.7357	9.6691
		320PB	-8.7667	4.46584	.202	-22.4691	4.9357
	320PB	220PB	4.7333	4.46584	.570	-8.9691	18.4357
		270PB	8.7667	4.46584	.202	-4.9357	22.4691
AFB1 %	220PB	270PB	-2.4333	6.72017	.931	-23.0527	18.1860
		320PB	-9.4000	6.72017	.399	-30.0193	11.2193
	270PB	220PB	2.4333	6.72017	.931	-18.1860	23.0527
		320PB	-6.9667	6.72017	.583	-27.5860	13.6527
	320PB	220PB	9.4000	6.72017	.399	-11.2193	30.0193
		270PB	6.9667	6.72017	.583	-13.6527	27.5860
AFB2 %	220PB	270PB	12.6000*	3.23854	.019	2.6633	22.5367
		320PB	10.7333*	3.23854	.037	.7966	20.6701
	270PB	220PB	-12.6000*	3.23854	.019	-22.5367	-2.6633
		320PB	-1.8667	3.23854	.837	-11.8034	8.0701
	320PB	220PB	-10.7333*	3.23854	.037	-20.6701	-.7966
		270PB	1.8667	3.23854	.837	-8.0701	11.8034
AFG1 %	220PB	270PB	-4.8333	10.19862	.886	-36.1255	26.4588
		320PB	3.7667	10.19862	.928	-27.5255	35.0588
	270PB	220PB	4.8333	10.19862	.886	-26.4588	36.1255
		320PB	8.6000	10.19862	.692	-22.6922	39.8922
	320PB	220PB	-3.7667	10.19862	.928	-35.0588	27.5255
		270PB	-8.6000	10.19862	.692	-39.8922	22.6922
AFG2 %	220PB	270PB	5.5667	6.55614	.689	-14.5494	25.6827
		320PB	2.1667	6.55614	.942	-17.9494	22.2827
	270PB	220PB	-5.5667	6.55614	.689	-25.6827	14.5494
		320PB	-3.4000	6.55614	.865	-23.5160	16.7160
	320PB	220PB	-2.1667	6.55614	.942	-22.2827	17.9494
		270PB	3.4000	6.55614	.865	-16.7160	23.5160
OTA %	220PB	270PB	4.5333	2.02777	.143	-1.6884	10.7551
		320PB	2.7333	2.02777	.423	-3.4884	8.9551
	270PB	220PB	-4.5333	2.02777	.143	-10.7551	1.6884
		320PB	-1.8000	2.02777	.667	-8.0218	4.4218
	320PB	220PB	-2.7333	2.02777	.423	-8.9551	3.4884
		270PB	1.8000	2.02777	.667	-4.4218	8.0218
ZEA %	220PB	270PB	8.0667*	2.46471	.039	.5042	15.6291
		320PB	5.8667	2.46471	.119	-1.6958	13.4291
	270PB	220PB	-8.0667*	2.46471	.039	-15.6291	-.5042
		320PB	-2.2000	2.46471	.664	-9.7624	5.3624
	320PB	220PB	-5.8667	2.46471	.119	-13.4291	1.6958
		270PB	2.2000	2.46471	.664	-5.3624	9.7624

Based on observed means.

The error term is Mean Square (Error) = 9.112.

*. The mean difference is significant at the .05 level.

Tukey HSD (Uses Harmonic Mean Sample Size = 3, alpha = .05)

DON %

Case	N	Subset 1
270PB	3	77.0000
220PB	3	81.0333
320PB	3	85.7667
Sig.		.202

Means for groups in homogeneous subsets are displayed.
Based on observed means.
The error term is Mean Square (Error) = 29.916.

AFB1 %

Case	N	Subset 1
220PB	3	52.2000
270PB	3	54.6333
320PB	3	61.6000
Sig.		.399

Means for groups in homogeneous subsets are displayed.
Based on observed means.
The error term is Mean Square (Error) = 67.741.

AFB2 %

		Subset	
Case	N	1	2
270PB	3	69.2333	
320PB	3	71.1000	
220PB	3		81.8333
Sig.		.837	1.000

Means for groups in homogeneous subsets are displayed.
Based on observed means.
The error term is Mean Square (Error) = 15.732.

AFG1 %

Case	N	Subset 1
320PB	3	55.5000
220PB	3	59.2667
270PB	3	64.1000
Sig.		.692

Means for groups in homogeneous subsets are displayed.
Based on observed means.
The error term is Mean Square (Error) = 156.018.

AFG2 %

Case	N	Subset 1
270PB	3	62.5000
320PB	3	65.9000
220PB	3	68.0667
Sig.		.689

Means for groups in homogeneous subsets are displayed.
Based on observed means.
The error term is Mean Square (Error) = 64.474.

OTA %

Case	N	Subset
		1
270PB	3	97.8000
320PB	3	99.6000
220PB	3	102.3333
Sig.		.143

Means for groups in homogeneous subsets are displayed.
Based on observed means.
The error term is Mean Square (Error) = 6.168.

ZEA %

Case	N	Subset	
		1	2
270PB	3	74.1333	
320PB	3	76.3333	76.3333
220PB	3		82.2000
Sig.		.664	.119

Means for groups in homogeneous subsets are displayed.
Based on observed means.
The error term is Mean Square (Error) = 9.112.
